# Genomic Landscape Analysis of Canine Pulmonary Adenocarcinoma Reveals Candidate Targetable Gene Fusions

**DOI:** 10.1111/vco.70063

**Published:** 2026-03-31

**Authors:** Sharadha Sakthikumar, William P. D. Hendricks, David Rainford, William Selleck, Natalia Briones, Christopher Coggins, Natalie Quan, Victoria Zismann, Gwendolen Lorch, Aleksandar Sekulic, Jeffrey M. Trent

**Affiliations:** ^1^ Translational Genomics Research Institute Phoenix Arizona USA; ^2^ MedVet Columbus Ohio USA

**Keywords:** cancer, cPAC, dogs, fusions, NSCLC, pulmonary adenocarcinoma

## Abstract

Spontaneously occurring primary canine pulmonary adenocarcinoma (cPAC) exhibits clinicopathological and molecular similarities to never‐smoker human lung cancers. Shared genomic alterations, including point mutations, indel mutations and copy number changes particularly in HER2 signalling, are significant therapeutic targets, especially for HER2 and tyrosine kinase inhibitors. Whilst progress has been made in identifying mutational drivers in canine cancers, the role of somatic gene fusions in cPAC remains poorly understood, despite their importance in other cancers as drivers and therapeutic targets. This study investigates the fusion landscape in cPAC by analysing RNA‐seq data from a cohort of 36 primary tumour samples and reports oncogenic fusions with therapeutic potential. Notably, NRG1 fusions were identified in a subset of tumours, including recurrent SDC4::NRG1 events, potentially playing key roles in disease progression. NRG1 fusions, known to activate HER2 signalling, are mutually exclusive with HER2 gene alterations, indicating convergence on the same pathway. Tumours with SDC4::NRG1 fusions also overexpress HER2 pathway‐related genes, reinforcing NRG1‐driven activation. Similar fusions occur in never‐smoker human non‐small cell adenocarcinoma lacking other common drivers, underscoring their therapeutic importance. These findings highlight NRG1 fusions as critical contributors to cPAC tumorigenesis and warrant further clinical and comparative investigation. Additionally, novel fusions disrupting the PTEN axis were identified, leading to truncated PTEN and associated protein domains. These disruptions could impair tumour‐suppressive pathways, presenting additional therapeutic targets. This research emphasises the broader relevance of fusion‐driven mechanisms in cPAC tumorigenesis, advancing the understanding of both canine and human lung cancers for clinical and comparative studies.

## 1 Introduction

Lung cancer is one of the most prevalent cancers in humans [1], with non‐small cell lung cancer (hNSCLC) being the most common type, accounting for 85% of all cases [1]. In dogs, lung cancer is comparatively rare, making up only 1% of all canine cancers [2]. The canine equivalent of hNSCLC is canine pulmonary adenocarcinoma (cPAC), which has an annual incidence rate of 15 per 100 000 dogs in the United Kingdom [3]. Although large‐scale epidemiological studies evaluating breed predisposition to cPAC have not been conducted, one study of 88 dogs reported that mixed breeds were more frequently represented (25%) than purebreds, including Labrador Retrievers (21%) and Bichon Frise (5%) [4]. Despite its rarity, cPAC shares similar clinical features, histopathological characteristics and oncogenic mechanisms with human lung cancer [5]. Moreover, the disease trajectory and biology of cPAC closely mirror those observed in hNSCLC [6].

Evidence suggests that environmental risk factors for lung cancer are shared across species. As companion and service animals, dogs often inhabit the same environments as humans, making them potential sentinels for human lung cancer risk factors. For instance, studies indicate that dogs with short (brachycephalic) or medium‐length (mesocephalic) noses, such as Labrador retrievers, may have an increased risk of developing cPAC if they live in homes with smokers (OR: 2.4; 95% CI, 0.7–7.8) [7]. Primary lung tumours in dogs, typically arising in older animals (median age 11 years), often resemble hNSCLC histotypes, including cPAC, canine pulmonary adenosquamous carcinoma (cPASC) and canine pulmonary squamous cell carcinoma (cPSCC) [8]. In one sequencing‐based study, papillary adenocarcinoma was the most commonly reported subtype, accounting for 62% of cases; however, this likely reflects the characteristics of the selected cohort and may not represent the true distribution of subtypes across the broader canine population [4].

Like human lung cancer, cPAC is frequently diagnosed late, often incidentally during routine geriatric examinations, evaluations following trauma or when nonspecific symptoms such as dyspnea (6%–24%) and cough (52%–93%) appear, usually only after the tumour has grown larger than 3 cm [9]. In a retrospective study of 40 dogs with primary lung tumours, median survival time was reported as 456 days for dogs with localised disease, but decreased significantly to 167 days when lymph node involvement was present, highlighting the prognostic impact of metastasis in canine pulmonary cancer [10].

The cPAC model represents a spontaneous, naturally occurring form of cancer that closely resembles hNSCLC in never‐smokers, highlighting shared molecular characteristics. These similarities include frequent mutations in key cancer‐related genes such as *HER2*, *CDKN2A*, *TP53*, *SMAD4* and *KRAS* [4]. For example, the *HER2* (also known as *ErbB2*) V659E mutation, found in 38% of cPAC cases, is orthologous to the ErbB2 V659E activation mutation reported in human lung adenocarcinoma [11], indicating significant molecular overlap with human lung cancer. In hNSCLC, these HER2‐activating aberrations are treatable with HER2‐targeted therapies [12]. Thus, identifying these HER2‐driven events in canine cancer, beyond the scope of currently recognised mutational homology, could provide valuable insights into the effectiveness of small‐molecule inhibitor therapies for HER2‐mutated cPAC.

In addition to HER2‐driven events, other somatic alterations, such as gene fusions, may drive oncogenesis in cPAC, similar to observations in hNSCLC [13]. Gene fusions result from structural DNA rearrangements, including translocations, insertions, transcription read‐throughs or splicing events [14]. These alterations are critical oncogenic triggers that can initiate disease or contribute to cancer progression, leading to the overexpression of driver oncogenes [15]. Oncogenic fusions are validated therapeutic targets in hNSCLC and are often clinically associated with a never‐smoking history, younger age and adenocarcinoma histology [16]. Analysis of the human cancer genome and transcriptome has identified hNSCLC rearrangements in fusion kinases such as *ROS1*, *ALK*, *RET*, *NTRK1* and *BRAF*, which often occur without concurrent driver mutations in *ErbB2*, *KRAS*, *BRAF*, *AKT* and *PIK3CA*. Whilst fusions involving *NRG1* are infrequent, their fusion partners are known to be clinically actionable in hNSCLC [17].

Although sporadic fusions have been observed in canine cancers, such as hematologic malignancies, gliomas and dermatofibrosarcomas [18], a systematic investigation to characterise fusions across various canine cancers, including pulmonary adenocarcinomas, remains largely unexplored. This gap underscores the need for further research, as a deeper understanding of these oncogenic fusions could reveal critical insights into disease mechanisms and potential therapeutic targets in canine cancers.

A thorough genome‐wide interrogation is needed to discover and characterise a broad spectrum of novel genetic alterations in cPAC, from point mutations to large structural variants. With improved reference assemblies [19, 20] and enhanced somatic detection algorithms now available, this study represents the first systematic investigation utilising these advancements to explore and uncover the oncogenic fusion landscape in canine cancer, specifically cPAC. We aimed to bridge this gap by employing comprehensive genomic and transcriptomic analyses alongside a new canine reference genome to systematically investigate novel somatic alterations that could have oncogenic and therapeutic significance in cPAC.

Here, we describe the genomic analysis of cPAC in 46 dogs using multi‐modal approaches, including next‐generation sequencing (NGS), quantitative PCR (qPCR), and Sanger methods to uncover critical insights into disease mechanisms and potential therapeutic targets, thereby advancing our understanding of cPAC and its parallels with human lung cancer. We have identified novel cPAC fusion and translocation events implicated in hNSCLC, highlighting their potential involvement in oncogenic processes and pathways. These findings pave the way for innovative treatment options and deeper insights into the shared genetic underpinnings of cancer across species.

## 2 Materials and Methods

### 2.1 Sample Collection

Tumour (T) tissues and, when available, matched normal (N) lung tissues were obtained from 46 client‐owned dogs previously diagnosed with pulmonary adenocarcinoma. These samples were collected during prior clinical procedures, immediately snap‐frozen in liquid nitrogen and stored at −80°C until analysis. Tumour diagnoses were confirmed by board‐certified veterinary pathologists (ACVP) through histopathological evaluation. This study involved a retrospective analysis of the archived samples. Ethical approval was granted by The Ohio State University Institutional Animal Care and Use Committee (IACUC; protocol number 2010A0015‐R2), and informed consent was obtained from all dog owners at the time of original sample collection.

### 2.2 Isolation and Sequencing of RNA and DNA From cPAC Cohort Samples

The current investigation involved whole‐genome sequencing (WGS), whole‐exome sequencing (WES), and RNA sequencing (RNA‐seq) of samples from a cPAC cohort. RNA and DNA were isolated using Qiagen AllPrep kits (catalogue ID: 80204) according to the manufacturer's protocol, and nucleic acid quality was assessed using the Thermo Fisher NanoDrop and Agilent TapeStation 2200 systems. Exome libraries for matched tumour‐normal (T‐N) samples were prepared with a custom Agilent SureSelect XT capture kit, which included 982 789 probes targeting 19 459 genes mapped to the canine reference genome (CanFam3.1) [21], covering approximately 43 *Mbp* of target sequence. Sequencing was performed on both tumour and normal samples: WGS libraries were sequenced on the Illumina NovaSeq 6000 platform, and WES libraries on the HiSeq 2000 platform. Target sequencing depths were 160× for WES samples and 60×/30× for tumour/normal WGS samples.

RNA libraries from tumour samples were prepared using TruSeq RNA Access (catalogue ID: 20020189) or Kapa RNA Hyper kits (catalogue ID: 08098107702). Libraries were sequenced on an Illumina NovaSeq 6000 to a target of 100 million reads per sample. The raw FASTQ format read files were trimmed for adapters and quality using fastp v0.23.4 (https://github.com/OpenGene/fastp). Read pairs were discarded if more than 40% of their bases had a Phred quality score below 15 or if the read length was shorter than 50 base pairs.

### 2.3 Alignment to Reference Assemblies

Alignment of WGS and WES trimmed FASTQ reads to the canine reference genome (CanFam4/UU_Cfam_GSD_1.0) [19] was performed with NVIDIA Parabricks fq2bam v4.0.1‐1 (GPU‐accelerated BWA v0.7.15) [22]. Although the exome capture design was based on CanFam3.1, sequencing reads were aligned and all downstream analyses were conducted using the more recent CanFam4 assembly to take advantage of its improved genome continuity and gene annotation. For the RNA‐seq reads, the tool Salmon v1.10.2 [23] was used to map them to the CanFam4 transcriptome assembly and estimate the abundance of the mapped transcripts.

### 2.4 Somatic Fusion Detection

STAR‐Fusion, a module of the Trinity Cancer Transcriptome Analysis Toolkit (CTAT) [24], was utilised to detect and profile fusion transcripts in the cohort. The initial step in executing the CTAT fusion workflow involved customising the list of known fusion transcripts in human cancer for the canine genome. This process involved extracting only those partners whose genes have been annotated in the canine reference assembly. Subsequently, a custom canine CTAT genome library was created, comprising data files such as the reference assembly, its associated gene annotations, the fusion transcripts and other genomic features essential for identifying fusion events. The resulting CTAT genome library, ‘ctat_genome_lib_build_dir/’, along with paired RNA fastq sequencing reads corresponding to a given sample/tumour in the cohort, were specified as input parameters for the fusion detection module STAR‐Fusion, which was executed with default settings to detect fusions within the sample. In addition to the default filtering employed by the module, a cutoff of 10 or more reads for the ‘JunctionReadCount’ parameter was applied.

### 2.5 Somatic Point and Indel Mutation Detection

Somatic point mutations (SPM) and somatic insertion/deletion mutations (SIM) were called in both whole‐genome and exome samples using two callers: NVIDIA Parabricks mutectcaller v4.0.1‐1 (GPU‐accelerated Mutect2 v.4.2.0.0) [25], and Strelka v2.9.7 [26]. Default parameters and recommended best practises for filtering false positives were applied to all the tools. The resulting variant call format (VCF) files containing the SPM and SIM calls were normalised, left‐aligned and intersected using BCFTools v1.19 (https://github.com/samtools/bcftools).

The Variant Effect Predictor v111.0 (VEP) [27] was used to annotate the consequences and impact of filtered consensus SPM and SIM calls. The tool detailed the genes affected by the mutations and delineated their putative impacts, indicating whether they have a detrimental effect on the proteins encoded by the genes. The variation consequences were categorised as ‘HIGH’ for having a disruptive impact, ‘MODERATE’ for changing the effectiveness of the protein, ‘LOW’ assumed to be largely benign and ‘MODIFIER’ for non‐coding variants or variants whose impact could not be predicted. Subsequently, the annotated VCFs were further filtered to include only HIGH or MODERATE impact variants, as indicated by VEP.

### 2.6 Quantitative PCR and Sanger Sequencing Validation of SDC4::NRG1 Fusion

All samples underwent fusion validation using qPCR. For those samples identified as positive for the SDC4::NRG1 fusion, the recurrent fusion junction was verified using Sanger sequencing. Both qPCR and Sanger sequencing used the following primers covering each side of the fusion junction:

SDC4::NRG1 forward: 5′‐GGGATGAGGATGTATCTAACAAGGT‐3′.

SDC4::NRG1 reverse: 5′‐AAGCACTCGCCTCCATTC‐3′.

qPCR was performed on a Quantstudio 6 Flex qPCR platform according to the manufacturer's protocol using a KAPA SYBER FAST kit (catalogue ID: 07959397001). qPCR data was analysed using the Quantstudio Real‐Time PCR software v1.1. A Ct value of < 30 was defined as fusion‐positive, whereas samples with Ct values ≥ 30 were considered fusion‐negative. For Sanger sequencing, Universal M13 sequences were added to the qPCR primers amplified, and samples were sequenced at Arizona State University's Genomics Core Facility (https://cores.research.asu.edu/genomics). Sanger sequencing results were visually inspected for the fusion breakpoint.

### 2.7 Differential Expression Analysis

Transcripts from RNA‐seq were length‐scaled and normalised to transcripts per million (TPM) using tximport v1.26.1 [28]. Subsequently, gene‐level counts were normalised to the library size across samples using edgeR v.3.40.2 [29]. Differential expression analysis was performed on the RNA‐seq data using limma v3.54.2 [30] with a four‐condition experimental model. Differentially expressed genes (DEGs) were filtered based on significance (*p*‐value < 0.05), fold change (absolute log fold change ≥ 1), and expression level (Average Expression > 1) thresholds.

## 3 Results

### 3.1 Multi‐Modal Sequencing of cPAC Cohort

We investigated somatic alterations in a cohort of 46 cPAC cases. The cohort ranged in age from 7 to 15 years, with a median age of 11 years. It comprised 19 spayed females, 25 neutered males and one intact male. The sex of one dog was unknown. (Table S1).

Matched tumour and normal samples were sequenced using whole‐genome (WGS, *n* = 8), whole‐exome (WES, *n* = 5) and RNA sequencing (*n* = 36, tumour‐only) approaches. Some samples underwent multiple sequencing methods (Table S1, Figure S1). The WGS and WES data were aligned to the CanFam4 reference genome [19], and passed quality thresholds, with median sequencing depths of 71× (range: 49–80) for WGS tumour and 41× (range: 13–65) for WGS normal samples, and 96× (range: 66–171) for WES tumour and 96× (range: 44–172) for WES normal samples. RNA sequencing provided a median of 4.4 million reads per sample (ranging from 0.91 to 25.3 million), covering 20 668 genes in the transcriptome.

### 3.2 Novel and Known Human Lung Cancer Gene Fusions Identified in cPAC

**FIGURE 1:**
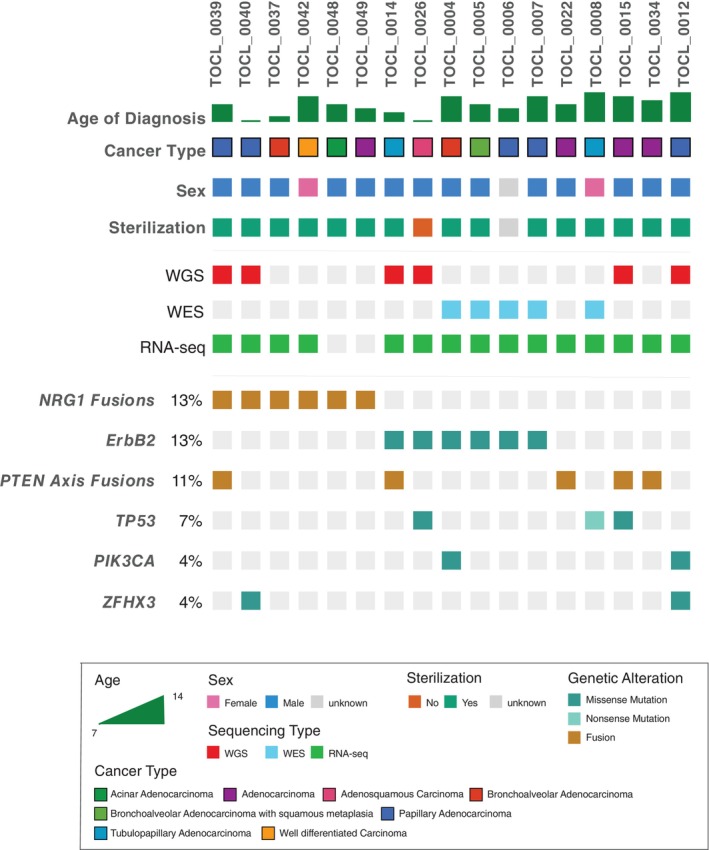
Oncoprint displays the distribution of genes altered in at least two samples in the cohort, along with their biological metadata. The oncoprint shows 17 samples, with each vertical bar representing a single patient, showing their age at diagnosis (top track) and the specific subtype of cPAC they are affected by. The somatically altered genes and their alteration frequency (calculated relative to the entire cohort of 46 tumours) are denoted on the left. Please refer to the legend inset for an explanation of the colour codes.

STAR‐Fusion identified 26 gene fusions in 19 (53%) of the tumours from 36 dogs with RNA‐seq data (Table 1, Figure 1). Notable events featured recurrent NRG1 fusions, with SDC4::NRG1 (3/36, 8%) and the singleton PTN::NRG1, both of which have been previously reported in hNSCLC and other cancers [31]. Additionally, the MAGI2::TNFRSF1B fusion was found in three tumours. *MAGI2*, a member of the MAGUK family of scaffolding proteins, is known to interact with the tumour suppressor *PTEN*, functioning as a scaffolding protein that stabilises PTEN protein and enhances its tumour‐suppressive effects [32]. Together with PTEN::ADGRL3 and PTEN::SCARB1 fusions found in two other tumours, these findings suggest the potential involvement of the PTEN signalling axis in cPAC.

**TABLE 1:** Comprehensive overview of fusions discovered in the cPAC cohort.

Index	Sample ID	Left gene	Left gene breakpoint	Right gene	Right gene breakpoint	Chimeric protein	Fusion type	Rearrangment type	Known or novel fusion?	Left gene cancer role	Right gene cancer role	Junction depth	HER2 (ErbB2) V659E mutual exclusivity/co‐occurence
1	TOCL_0024	ADAM9	chr16:27196439	ATG4C	chr5:47679640	ADAM9::ATG4C	Tail–Tail	CTX	Novel	Fusion partner	Fusion partner	18	Mutually exclusive
2	TOCL_0028	ASPH	chr29:12329231	SNTG1	chr29:2576510	ASPH::SNTG1	Tail–Head	ITX	Novel	Fusion partner	Fusion partner	20	Mutually exclusive
3	TOCL_0021	CADPS2	chr14:60402732	SIRT7	chr9:1203435	CADPS2::SIRT7	Tail–Head	CTX	Novel	Fusion partner	Fusion partner	28	Mutually exclusive
4	TOCL_0042	CNOT4	chr16:12383469	PTN	chr16:11027863	CNOT4::PTN	Head–Head	ITX	Novel	Fusion partner	Fusion partner	42	Mutually exclusive
5	TOCL_0038	CTCF	chr5:82410083	MRTFB	chr6:29236753	CTCF::MRTFB	Tail–Tail	CTX	Novel	TSG	Fusion partner	122	Mutually exclusive
6	TOCL_0047	FRS2	chr10:11857575	PTPRB	chr10:12710233	FRS2::PTPRB	Head–Tail	ITX	Known	ONC	TSG	175	Mutually exclusive
7	TOCL_0021	GNAI2	chr20:39502007	BCL2L11	chr17:35812807	GNAI2::BCL2L11	Tail–Head	CTX	Novel	Fusion partner	TSG	15	Mutually exclusive
8	TOCL_0014	INVS	chr11:57393072	ASTN2	chr11:70900000	INVS::ASTN2	Head–Tail	ITX	Novel	Fusion partner	Fusion partner	16	Co‐occurrence
9	TOCL_0014	MAGI2	chr18:18699870	TNFRSF1B	chr2:83618740	MAGI2::TNFRSF1B	Tail–Tail	CTX	Novel	TSG	ONC	25	Co‐occurrence
10	TOCL_0022	MAGI2	chr18:18699870	TNFRSF1B	chr2:83618740	MAGI2::TNFRSF1B	Tail–Tail	CTX	Novel	TSG	ONC	69	Mutually exclusive
11	TOCL_0039	MAGI2	chr18:18699870	TNFRSF1B	chr2:83618740	MAGI2::TNFRSF1B	Tail–Tail	CTX	Novel	TSG	ONC	11	Mutually exclusive
12	TOCL_0013	MTCL1	chr7:74928845	PTPRM	chr7:74643042	MTCL1::PTPRM	Head–Head	ITX	Novel	Fusion partner	Fusion partner	29	Mutually exclusive
13	TOCL_0026	MTHFD2	chr17:49385194	CTNNA2	chr17:43995654	MTHFD2::CTNNA2	Tail–Tail	ITX	Novel	TSG/ONC	ONC	127	Co‐occurrence
14	TOCL_0029	PCSK7	chr5:16397670	CTDNEP1	chr5:32418918	PCSK7::CTDNEP1	Head–Tail	ITX	Novel	Fusion partner	Fusion partner	11	Mutually exclusive
15	TOCL_0034	PTEN	chr26:38142556	ADGRL3	chr13:54079425	PTEN::ADGRL3	Head–Head	CTX	Novel	TSG	Fusion partner	225	Mutually exclusive
16	TOCL_0015	PTEN	chr26:38190045	SCARB1	chr26:5334653	PTEN::SCARB1	Head–Head	ITX	Novel	TSG	Fusion partner	46	Mutually exclusive
17	TOCL_0042	PTN	chr16:11031477	NRG1	chr16:32480596	PTN::NRG1	Head–Tail	ITX	Novel	Fusion partner	ONC/TSG/FUS	69	Mutually exclusive
18	TOCL_0037	SDC4	chr24:33265375	NRG1	chr16:32480596	SDC4::NRG1	Tail–Tail	CTX	Known	FUS	ONC/TSG/FUS	854	Mutually exclusive
19	TOCL_0039	SDC4	chr24:33265375	NRG1	chr16:32480596	SDC4::NRG1	Tail–Tail	CTX	Known	FUS	ONC/TSG/FUS	1106	Mutually exclusive
20	TOCL_0040	SDC4	chr24:33265375	NRG1	chr16:32480596	SDC4::NRG1	Tail–Tail	CTX	Known	FUS	ONC/TSG/FUS	748	Mutually exclusive
21	TOCL_0048	SDC4	chr24:33265375	NRG1	chr16:32480596	SDC4::NRG1	Tail–Tail	CTX	Known	FUS	ONC/TSG/FUS	1106	Mutually exclusive
22	TOCL_0049	SDC4	chr24:33265375	NRG1	chr16:32480596	SDC4::NRG1	Tail–Tail	CTX	Known	FUS	ONC/TSG/FUS	748	Mutually exclusive
23	TOCL_0021	SIRT7	chr9:1201437	METTL23	chr9:4936811	SIRT7::METTL23	Head–Tail	ITX	Novel	Fusion Partner	Fusion Partner	67	Mutually exclusive
24	TOCL_0020	SMIM7	chr20:46315220	LRRC36	chr5:82618458	SMIM7::LRRC36	Head–Tail	CTX	Novel	Fusion partner	Fusion partner	11	Mutually exclusive
25	TOCL_0022	SNX13	chr14:32339606	SGCE	chr14:20053113	SNX13::SGCE	Tail–Tail	ITX	Novel	Fusion partner	Fusion partner	13	Mutually exclusive
26	TOCL_0021	SUDS3	chr26:15057645	KSR2	chr26:14639835	SUDS3::KSR2	Head–Tail	ITX	Novel	Fusion partner	ONC	10	Mutually exclusive

Key for the columns		
Column no.	Attribute	Description
1	Index	Serial number of the fusion event.
2	Sample ID	Unique identifier for the tumour sample where the fusion was detected.
3	Left gene/right gene	Genes involved in the fusion; “left” and “right” refer to their position and orientation in the fusion transcript.
4	Left gene breakpoint/right gene breakpoint	Genomic coordinates (CanFam4 chromosome and position) where the break occurred in each gene.
5	Chimeric protein	Name of the resulting fusion protein.
6	Fusion type	Orientation of the fused genes: Tail–Head, Tail–Tail, Head–Head, or Head–Tail.
7	Rearrangement type	Type of genomic rearrangement: CTX = interchromosomal translocation; ITX = intrachromosomal translocation.
8	Known or novel fusion?	Indicates whether the fusion has been previously reported in databases or the literature.
9	Left/right gene cancer role	Functional role of each gene in cancer (ONC = oncogene; TSG = tumour suppressor; FUS = fusion gene; fusion partner = reported in literature).
10	Junction depth	Number of sequencing reads supporting the fusion junction.
11	HER2 V659E mutual exclusivity/co‐occurrence	Indicates whether the fusion and HER2 V659E mutation are mutually exclusive or co‐occurring in the same sample.

Abbreviation	Description
ITX	Intra‐chromosomal
CTX	Inter‐chromosomal
ONC	Oncogene
TSG	Tumour suppresor gene
FUS	Fusion gene

We also identified 20 novel fusion transcripts not previously reported in human cancers [31]. These involve genes linked to tumour suppression (*MAGI2*, *MRTFB*, *PTEN*) and oncogenesis (*CTNNA2*). Nearly all genes in these fusions have been associated as fusion partners in various human malignancies (Table S2).

The SDC4::NRG1 fusion, known for its role in hNSCLC, was prioritised for validation. Experimental confirmation was achieved in 5 of 46 tumours (11%), including three identified by RNA‐seq, using both qPCR and Sanger sequencing (results not shown). Fusion‐specific primers amplified products exclusively in fusion‐positive samples, with Ct values ranging from 15.8 to 28.8, whilst no amplification was observed in negative controls (Table S3), underscoring the fusion's relevance in this cPAC cohort.

### 3.3 Features and Attributes of the Fusion Partners

**FIGURE 2:**
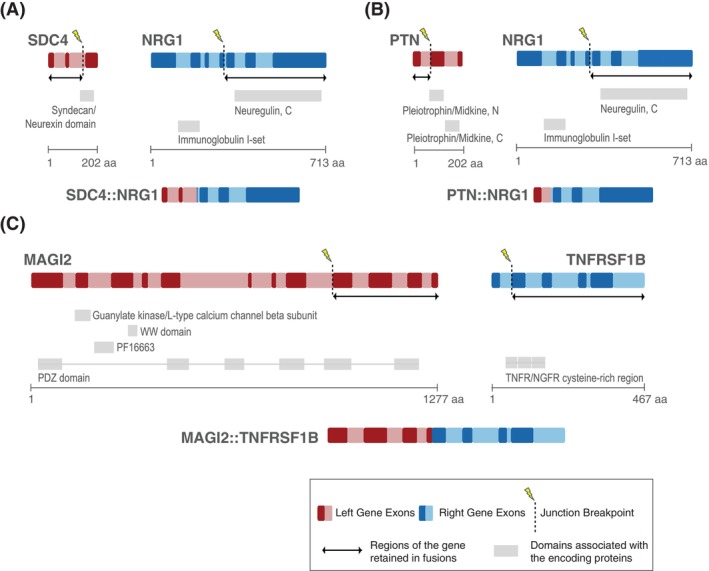
Recurrent fusions seen in canine pulmonary adenocarcinoma. (A) Inter‐chromosomal translocation between SDC4 and NRG1 forms the SDC4::NRG1 fusion protein, retaining the Neuregulin domain from NRG1 and part of the Syndecan domain from SDC4. (B) Intra‐chromosomal translocation between PTN and NRG1 results in the PTN::NRG1 fusion protein, in which the Neuregulin domains from NRG1 are retained, and the N and C domains of PTN/MK are completely excised from the final product. (C) Inter‐chromosomal translocation between MAGI2 and TNFRSF1B produces the MAGI2::TNFRSF1B fusion, with all MAGI2 domains deleted, rendering it inert, whilst TNFRSF1B retains its domains and potential activity.

The SDC4::NRG1 fusion product results from the abnormal fusion of the *SDC4* and *NRG1* genes in a tail‐to‐tail orientation, positioning SDC4 as the left and NRG1 as the right partner (Figure 2A). Specific breakpoints in each gene lead to distinct patterns of retention and excision for various domains. In the SDC4‐encoded protein, the Syndecan (SD4) domain is partially deleted, whilst in NRG1, the hEGF and Neuroglin domains are retained and the I‐Set domain is excised. As a result, the final fusion protein includes two intact functional domains from NRG1 and one partially truncated domain from SDC4 (Figure 2A).

The PTN‐NRG1 fusion forms similarly to SDC4::NRG1 but occurs intra‐chromosomally and in a head‐to‐tail configuration (Figure 2B). NRG1 fragments and fuses with PTN at loci homologous to those in the SDC4::NRG1 fusion, retaining the same protein domains. However, both the PTN/MK N and PTN/MK C domains are excised from PTN, likely rendering the PTN segment of the fusion protein functionally inactive/inert.

The MAGI2::TNFRSF1B fusion results from a tail‐to‐tail fusion of the *MAGI2* and *TNFRSF1B* genes (Figure 2C). In MAGI2, this fusion deletes the PDZ domain, although the N‐terminal sequence remains intact, potentially impairing its capacity for protein interactions. In contrast, the TNFRSF1B partner retains its TNFR domain, indicating that its signalling functions may be preserved, which could confer oncogenic potential.

Lastly, both PTEN fusion products—PTEN::ADGRL3 and PTEN::SCARB1—lead to truncated PTEN proteins that lack the essential phosphatase domain (Figure S2A,B). In PTEN::ADGRL3, the SUEL lectin and olfactomedin‐like domains are deleted, impacting adhesion and signalling functions. Conversely, in PTEN::SCARB1, the CD36 domain remains intact, likely supporting roles in endocytosis and metabolism [21].

### 3.4 ErbB2 Mutations Exhibit Mutual Exclusivity With the Fusions in the Cohort

Analysis of WES and WGS sequence data from 13 dogs using the intersect and combine approach with Mutect2 and Strelka revealed 791 SPM and 31 SIM calls across all individuals. VEP was used to annotate these variants, detecting protein‐modifying changes (either high‐ or moderate‐impact) in 614 genes (Figure 1, Table S4). Amongst these, 24 genes exhibited alterations in two or more tumours. Notably, *ErbB2* emerged as the most frequently mutated gene, with the missense mutation ErbB2 V659E observed in five tumours and K676E in one, consistent with previous findings [4] using the CanFam3.1 reference assembly. In addition, except for three sequenced tumours, there was mutual exclusivity between the discovered fusions and the above putative driver *ErbB2* mutation.

Additionally, VEP predicted high‐impact mutations in key tumour suppressor genes (e.g., *TP53*) and oncogenes such as *PIK3CA* and *KRAS*, amongst others (Table S4). A total of 18 genes identified as mutated in our cohort, including those mentioned above, are known to be significantly mutated in human lung cancer (Table S4) [33].

### 3.5 ErbB and JAK–STAT Signalling are Upregulated in cPAC

**FIGURE 3:**
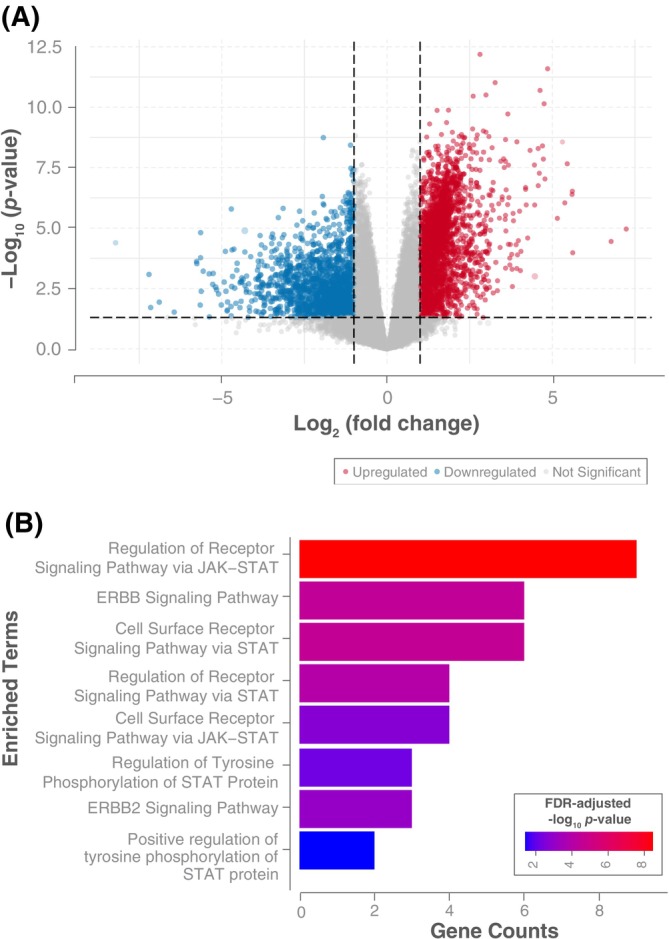
(A) Volcano plots comparing the expression of genes in NRG1‐mutated and wildtype cPAC samples. The genes with a log_2_ fold change greater than 1 or less than −1 (indicated by vertical black dashed lines) and *p*‐values less than 0.05 (indicated by a horizontal black dashed line) are coloured. Blue points represent significantly downregulated genes, whilst red points represent significantly upregulated genes. Grey points indicate genes that do not meet these significance thresholds. (B) Implicated canonical enrichment terms in cPAC. The bar chart displays the significantly enriched GO Terms as predicted by ENRICHR using NRG1^mut^ versus NRG1^wt^ differentially expressed genes. The bar colours reflect the −log_10_ of the adjusted *p*‐values for the enrichment scores, with red indicating more significant terms (lower adjusted *p*‐values) and blue indicating less significant ones (higher adjusted *p*‐values).

To understand how genes and pathways are differentially expressed under various conditions, we performed differential expression analyses (DEA; Figure S3). Conditions 1, 2 and 4 all yielded differentially expressed genes (DEGs); however, only Condition 1, comparing SDC4::NRG1 fusion‐positive samples to NRG1‐wild‐type samples, revealed significant enrichment in oncogenic pathways relevant to the disease. This comparison identified 1720 significantly DEGs (adjusted *p*‐value < 0.05), comprising 938 upregulated and 782 downregulated genes (Figure 3A, Table S5). Gene ontology (GO) enrichment analysis, with DEGs implicated in cancer [31], revealed significant enrichment in pathways such as ErbB signalling, JAK–STAT signalling, and IL‐6 signalling (Figure 3B, Table S6) in the SDC4::NRG1 fusion‐containing samples. The set is linked to several enriched Kyoto Encyclopaedia of Genes and Genomes (KEGG) pathways, including non‐small cell lung cancer (Table S7).

## 4 Discussion

Gene fusions are well‐established drivers in many human cancers; however, efforts to identify and characterise fusion events in canine tumours have been sporadic. Early discoveries relied on fluorescence in situ hybridization (FISH), which enabled the detection of oncogenic rearrangements such as the BCR::ABL fusion in canine leukaemia [34]. A methodological shift occurred with the introduction of RNA sequencing to canine tumours, expanding the scope from targeted, case‐specific detection to broad, transcriptome‐wide discovery. This approach revealed that dogs with B‐cell lymphoma, glioma and dermatofibrosarcoma‐like tumours harboured fusions with conserved breakpoints and oncogenic potential [18]. More recently, an RNA‐seq study in canine hemangiosarcomas identified novel, subtype‐specific fusions that frequently co‐occurred with *TP53* mutations, suggesting a link between genomic instability and fusion‐driven angiogenic programmes [35]. Previously, high‐throughput fusion analyses in canines were constrained by limited access to RNA‐seq technologies, underdeveloped reference genomes, and a lack of specialised computational tools. However, recent advances in genome assemblies, transcriptomic infrastructure and fusion detection algorithms now enable the systematic discovery of gene fusions in canine cancers.

In the current investigation, through multi‐platform sequencing and validation, we identified novel and recurrent gene fusions in cPAC, many of which are likely contributing to oncogenesis. Notably, we detected fusions involving the NRG1 (Neuregulin 1) gene, part of the ErbB receptor ligand family. NRG1 fusions, previously reported in hNSCLC, suggest a conserved oncogenic mechanism across species. Furthermore, these fusions have been established as diagnostic biomarkers [13] and are considered oncogenic drivers, likely promoting both cancer initiation and maintenance [36]. They are also prognostic biomarkers associated with poor survival in hNSCLC [37]. In a study of Asian non‐smoking women with hNSCLC, the recurrent orthologous SDC4::NRG1 fusion, which we also observed in our canine cohort, was identified in tumours lacking other common driver mutations [16]. This finding suggests the SDC4::NRG1 fusion could serve as a potential therapeutic target in cases where conventional drivers are absent. Subsequent sections here delve into possible mechanisms of tumorigenesis mediated by SDC4::NRG1, including the aberrant activation of downstream ErbB family signalling pathways that regulate cell proliferation and survival.

Another key signalling pathway—the PTEN signalling axis—may also play a critical role in cPAC. Key PTEN gene fusions, such as PTEN::ADGRL3 and PTEN::SCARB1, may drive cancer progression by producing truncated PTEN proteins that lack the phosphatase domain essential for regulating the PI3K/Akt pathway [38]. This truncation is linked to increased cell proliferation and reduced apoptosis [39]. Specifically, PTEN::ADGRL3 disrupts cell–cell adhesion and GPCR signalling, which may enhance metastasis [40]. In PTEN::SCARB1, the retained CD36 domain could impact lipid metabolism, cholesterol uptake and immune response, contributing to tumour growth [41, 42]. These fusions are promising therapeutic targets, with opportunities to intervene by modulating GPCR signalling in PTEN::ADGRL3 or addressing metabolic dysregulation in PTEN::SCARB1.

Further oncogenic potential is suggested by fusions involving the PTEN stabiliser *MAGI2*. The MAGI2::TNFRSF1B fusion indicates oncogenic activity, impairing PTEN stabilisation and promoting pro‐survival signalling. This fusion lacks the MAGI2 PDZ domain, critical for stabilising PTEN and regulating Akt signalling. As a result, PTEN activity is likely reduced, leading to increased cell proliferation [43]. Meanwhile, the retained TNFR domain of TNFRSF1B (also known as *TNFR2*) enables sustained NFκB‐driven survival signalling, which has been associated with poor prognosis in various cancers [44]. The loss of PTEN regulatory function and persistent TNFR2 signalling likely drive oncogenesis, underscoring the MAGI2::TNFRSF1B fusion as a promising therapeutic target.

NRG1 encodes a versatile growth factor with an epidermal growth factor (EGF)‐like domain that activates ErbB3/4 receptors, key elements in the ErbB signalling network associated with cellular growth, division and proliferation. This interaction facilitates heterodimerization with other ErbB receptors, triggering downstream signalling cascades [45]. Our data demonstrate that the SDC4::NRG1 fusion in canine models preserves the EGF‐like domain, which is crucial for engaging with ErbB3/4 receptors and effectively initiating ErbB signalling.

In parallel, we observed a significant upregulation of genes involved in ErbB signalling, including *NRG1*, *ErbB2* and *EGF*, in SDC4::NRG1 fusion‐positive samples compared to wildtype. Pathway analyses further highlighted the enrichment of ErbB and JAK/STAT signalling pathways in our gene set. The well‐conserved JAK/STAT cascade regulates gene transcription vital for immune responses and cellular growth [46]. Activation of this pathway, documented in hNSCLC, is linked to tumour proliferation and patient survival [47].

A prior investigation [4] revealed the presence of recurrent ErbB2 V659E hotspot mutation in cPAC. Unique within the ErbB family, ErbB2 lacks a specific binding ligand and engages in ligand‐independent homo‐ or hetero‐dimerization with other family members, thus initiating signalling [48]. The V659E mutations, located in the transmembrane domain, promote dimer stabilisation and constitutive activation of ErbB pathways. This suggests that ErbB2 V659E mutations may activate oncogenic pathways downstream of ErbB signalling, including JAK/STAT, PI3K/AKT and MAPK pathways, mirroring observations in hNSCLC, where such mutations function as activating drivers [49].

Intriguingly, in our cPAC cohort, there is mutual exclusivity between ErbB2 V659E mutations and NRG1 fusions, indicating distinct oncogenic mechanisms within the ErbB signalling framework may converge phenotypically. Whilst NRG1 fusions facilitate ErbB3/4 dimerization, enhancing JAK/STAT signalling through NRG1 binding and IL‐6 binding to its receptor (IL‐6R), ErbB2 V659E mutations primarily stabilise ErbB2 dimers. The increased expression of EGF and NRG1, coupled with elevated IL‐6 levels, suggests a favourable environment for extensive ErbB dimer formation, highlighting a potential mechanism driving oncogenesis and progression in cPAC through JAK/STAT signalling activation by two distinct pathways.

Somatic alterations, gene expression patterns, and certain molecular pathways are conserved between cPAC and hNSCLC, equivalent lung malignancies. This conservation suggests therapeutic targets identified in human oncology may provide valuable insights into possible treatment approaches for canine tumours. Notably, there is compelling evidence of therapeutic potential in drugs targeting the ErbB and JAK/STAT signalling pathways in hNSCLC [50].

To date, no established cPAC therapies target either ErbB or JAK/STAT signalling. However, there is potential for repurposing human oncological drugs for canine patients. Tyrosine kinase inhibitors (TKIs) represent a category of targeted treatments that inhibit the activity of the ErbB family of receptors, including ErbB2, ErbB1 and ErbB4 [51]. For example, afatinib, an irreversible TKI, forms a covalent bond with HER1, HER2 and HER4 receptors, demonstrating efficacy in clinical settings [52]. In a cohort study, five patients with NRG1 fusion‐driven lung tumours, including those with SDC4::NRG1 fusions, experienced temporary cessation or regression of disease progression and stabilisation of their condition when administered afatinib [53]. Furthermore, another case study with afatinib reported clinical improvement and progression‐free survival of 6.5 months in a patient with hNSCLC [54]. The highlighted cases of hNSCLC treated with afatinib suggest that this drug could be administered to dogs with cPAC, although the response and tolerability may be limitations to consider.

**FIGURE 4:**
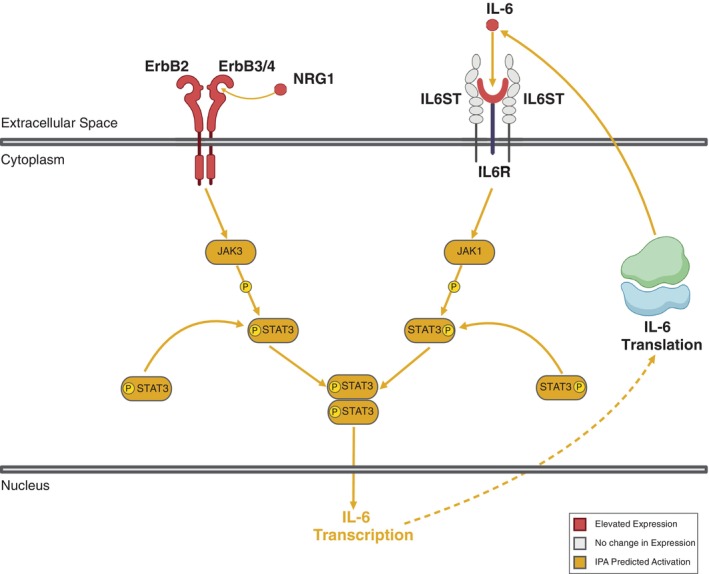
Schematic representation of ErbB and IL families signalling and their potential downstream cascades. On the left, NRG1 binds to ErbB3/4, which dimerizes ErbB3/4 to ErbB2, activating the JAK3‐mediated phosphorylation of STAT3. On the right, IL‐6 binds to the receptors of IL6R, which causes the JAK1‐mediated phosphorylation of STAT3. Both these processes can result in a positive feedback loop of IL‐6 transcription, translation and binding. JAK/STAT Janus kinase/signal transducer and activator of transcription. Image rendered using Biorender.

As outlined in the preceding sections, the ErbB signalling pathway enhances JAK/STAT activity through JAK3 phosphorylation of STAT3/5. TKIs represent a potential strategy for inhibiting this mechanism in cPAC; however, the JAK/STAT pathway can still be activated by binding IL‐6 to IL6R and subsequent phosphorylation of STAT3 by JAK1 (Figure 4). Ruxolitinib, a JAK1/2 inhibitor, obstructs the functional mechanisms of these enzymes and is conventionally used to treat myelofibrosis [55]. Cell viability assays have demonstrated that a combination of ruxolitinib and afatinib effectively kills hNSCLC cells [56]. The synergistic impact of ruxolitinib and afatinib was further examined in a phase Ib clinical trial (NCT02145637) involving patients with EGFR‐mutant, Stage IV hNSCLC. This combination treatment was well‐tolerated, eliciting a partial response in some trial participants [57]. The clinical evidence regarding the use of JAK and ErbB inhibitors in hNSCLC suggests that further investigation into their individual or combined application in NRG1 fusion‐positive tumours is warranted.

In conclusion, our study reveals the significance of *NRG1* fusions and *ErbB2* mutations in driving ErbB signalling in cPAC. NRG1 fusions, particularly the SDC4::NRG1 fusion, enhance ErbB signalling by promoting the dimerization of ErbB receptors, initiating downstream signalling pathways crucial for tumour growth and proliferation. Furthermore, differential expression analysis highlighted the upregulation of genes involved in ErbB and JAK/STAT signalling pathways in SDC4::NRG1 fusion‐positive samples, indicating a potential role for JAK/STAT signalling in cPAC oncogenesis. Targeting pathways, conserved between cPAC and hNSCLC, could offer therapeutic strategies for cPAC. Drugs targeting ErbB and JAK/STAT signalling, such as afatinib and ruxolitinib, have shown efficacy in hNSCLC and could be agents considered for cPAC clinical trials. Additionally, other molecular alterations, including novel fusions in the PTEN‐axis, highlight the complexity of cPAC's molecular landscape and provide more potential targets for therapeutic intervention.

These findings lay the groundwork for preclinical and clinical studies to explore targeted therapies, which could ultimately improve outcomes for canine cancer patients and contribute to advancements in comparative oncology.

## Funding

This study was supported by the National Canine Cancer Foundation (GL150H‐005; G. Lorch), philanthropic support to the TGen Foundation (J. Trent and W. Hendricks), National Institutes of Health, National Center for Advancing Translational Sciences (UL1TR001070; G. Lorch), National Cancer Institute (P30 CA016058; G. Lorch, J. Trent, and W. Hendricks), Brooke's Blossoming Hope (J. Trent and W. Hendricks), and a grant investment from the Petco Love in partnership with the Blue Buffalo Foundation for Cancer Research Inc.

## Conflicts of Interest

The authors declare no conflicts of interest.

## Supporting information


**Figure S1:** UpSet plot representing the overlap between whole genome sequencing (WGS), whole exome sequencing (WES), amplicon sequencing, RNA sequencing, qPCR, and sanger sequencing for the study cohort. Visualisation of overlap amongst sequencing and validation methods across samples in the study cohort.

**Figure S2:** Fusions involving the PTEN show deletion of the phosphatase domain in the encoding protein, leading to its dysregulation. Structural representation of PTEN fusion events highlighting deletion of the phosphatase domain in the resulting protein.

**Figure S3:** Schematic workflow of DEA across molecular subgroups. Each node represents a pairwise comparison, with the total number of biological replicates indicated per condition. Diagram illustrating the differential expression analysis workflow and pairwise comparisons across molecular subgroups.

**Table S1:** cPAC cohort clinical metadata, sequencing information and associated metrics.
**Table S2:** Number of distinct human cancers in which genes from cPAC fusion events have been reported in fusion events.
**Table S3:** qPCR confirmation of SDC4::NRG1 fusions.
**Table S4:** Summary of somatic point and indel mutations in the cPAC cohort.
**Table S5:** Differential expression of NRG1 fusion versus NRG1 wt tumours.
**Table S6:** Gene ontology enrichment of DEGs in NRG1 fusion samples.
**Table S7:** KEGG pathway enrichment of DEGs in NRG1 fusion samples.

## Data Availability

The summary data supporting the findings of this study are available in the Supporting Information of this article. The sequencing data (WGS, WES, RNA‐seq) have been deposited in the Sequence Read Archive (SRA) under accession number PRJNA1294405.
